# Post-disaster health impact of natural hazards in the Philippines in 2013

**DOI:** 10.3402/gha.v9.31320

**Published:** 2016-05-17

**Authors:** Miguel Antonio Salazar, Arturo Pesigan, Ronald Law, Volker Winkler

**Affiliations:** 1 Institute of Public Health, Heidelberg University, Heidelberg, Germany; 2 World Health Organization Office in Sri Lanka, Colombo, Sri Lanka; 3 Health Emergency Management Bureau, Department of Health, Republic of the Philippines, Manila, Philippines

**Keywords:** disasters, syndromic surveillance, infectious disease, non-communicable diseases, injuries

## Abstract

**Background:**

In 2011, the Health Emergency Management Bureau (HEMB) created the Surveillance for Post Extreme Emergencies and Disasters (SPEED), a real-time syndromic surveillance system that allows the early detection and monitoring of post-disaster disease trends. SPEED can assist health leaders in making informed decisions on health systems affected by disasters. There is a need for further validation of current concepts in post-disaster disease patterns in respect to actual field data. This study aims to evaluate the temporal post-disaster patterns of selected diseases after a flood, an earthquake, and a typhoon in the Philippines in 2013.

**Methodology:**

We analyzed the 21 syndromes provided by SPEED both separately and grouped into injuries, communicable diseases, and non-communicable diseases (NCDs) by calculating daily post-disaster consultation rates for up to 150 days post-disaster. These were compared over time and juxtaposed according to the type of disaster.

**Results:**

Communicable diseases were found to be the predominant syndrome group in all three disaster types. The top six syndromes found were: acute respiratory infections, open wounds, bruises and burns, high blood pressure, skin disease, fever, and acute watery diarrhea.

**Discussion:**

Overall, the results aligned with the country's morbidity profile. Within 2 months, the clear gradation of increasing syndrome rates reflected the severity (flood<earthquake<typhoon) and magnitude of the disruption of the health system caused by the disasters. After 2 months, rates dropped, suggesting the beginning of the recovery phase. The most common syndromes can be addressed by measures such as providing for shelter, water, sanitation, hygiene, nutrition, and common health services.

**Conclusions:**

Most post-disaster syndromes may be addressed by prevention, early diagnosis, and early treatment. Health needs differ in response and recovery phases.

## Introduction

Between 2000 and 2014, the Philippines experienced 245 disasters caused by natural hazards (1). In 1999, the Department of Health formalized the existence of the Health Emergency Management Staff, later to be known as the Health Emergency Management Bureau (HEMB), responsible for providing policy development for health emergency response, preparedness, logistics, and health emergency information systems through an administrative order (2, 3).

In support of its mandate, the HEMB together with the World Health Organization (WHO) created the Surveillance for Post Extreme Emergencies and Disasters (SPEED) in 2011. SPEED provides a real-time syndromic surveillance tool during emergencies and disasters that allows the early detection and monitoring of disease trends (3). Syndromic surveillance, defined as the real-time gathering of health information using non-specific indicators for early identification of health threats, has been used by low- and middle-income countries to rapidly build national capacities in disease surveillance (4, 5). SPEED is a type of Early Warning Alert and Response Network (EWARN); however, compared to an EWARN, SPEED involves non-communicable diseases (NCDs), such as known diabetes, high blood pressure, and injuries (3, 6).

In 2013, the Philippines experienced five floods, eight storms in the form of tropical cyclones, and one earthquake (1). SPEED was activated in the following disasters due to natural hazards: the Luzon Flood, the Bohol Earthquake, and Typhoon Haiyan. The Luzon Flood was a result of Tropical Storm Trami enhanced by a southwest monsoon, which hit the Philippines in August. It affected 2.5 million people across five regions (7). The Bohol Earthquake was a 7.2 magnitude earthquake, which occurred on October 15 and affected 3.2 million people (8). Typhoon Haiyan, a category 5 tropical cyclone, made landfall on November 8 and affected 14 million people (9, 10). These three events reflect some of the most common natural hazards that have affected the Philippines in the past decade, namely, floods, typhoons, and earthquakes (1).

These different types of natural hazards have both similarities and differences in the health impact on populations. Floods have been characterized to have a predominance of water-related infectious diseases, such as diarrhea, due to water contamination and damage to water systems (11–13). Flooding could also increase endemic vector-borne diseases by introducing more breeding sites for mosquito vectors, and facilitate the transmission of diseases such as leptospirosis (11, 13). Earthquakes, on the other hand, are thought to characteristically cause a high loss of life and injuries due to falling debris or structural collapse. However, due to the displacement of populations to evacuation centers, outbreaks of endemic infectious diseases exacerbated by overcrowding may also occur (11, 14). Typhoons or tropical cyclones similar to a flood may increase the burden of vector-borne diseases due to an increased influx of water through rain and storm surges (11, 13).

There has been a push for the inclusion of NCDs in the disaster response and preparedness planning (15, 16). In high-income countries such as the United States, chronic diseases accounted for a significant number of consultations after hurricane Katrina and Rita (16). Even low- to middle-income countries like Pakistan experienced a considerable number of chronic conditions, such as diabetes and hypertension, in stationary health clinics after the Kashmir-Pakistan earthquake (17).

Disease surveillance through SPEED can assist health leaders in making informed decisions in the response, preparedness, and recovery phases of disasters (18–20). There is a need for further validation of current concepts in post-disaster disease patterns by actual field data, which the SPEED database of HEMB can provide. The aim of this study is to evaluate the temporal post-disaster patterns of selected diseases after a flood, an earthquake, and a typhoon using the SPEED database from 2013.

## Methodology

The following is a descriptive analysis of the SPEED database from 2013 limited to natural hazard records. It includes consultations of various syndromes and their corresponding diagnoses. The recorded syndromes were grouped into infectious or communicable diseases, injuries, and NCDs (see Table 1) (3).

**Table 1:** SPEED syndromes

#	Syndrome	Initial diagnosis	Syndrome group
1	Difficulty of breathing and wheezing	Acute asthmatic attack	Non-communicable
2	Loose stools with visible blood	Acute bloody diarrhea	Communicable
3	Floppy paralysis of the limbs which occurred recently in a child <15 years who was previously normal	Acute flaccid paralysis	Communicable
4	Fever with spontaneous bleeding	Acute hemorrhagic fever	Communicable
5	Yellow eyes or skin with or without fever	Acute jaundice syndrome	Communicable
6	Visible wasting, with or without bipedal pitting edema	Acute malnutrition	Non-communicable
7	Cough, colds or sore throat with or without fever	Acute respiratory infection	Communicable
8	Loose stools, 3 or more in the past 24 h with or without dehydration	Acute watery diarrhea	Communicable
9	Animal bites	Animal bites	Communicable
10	Eye itchiness, redness with or without discharge	Conjunctivitis	Communicable
11	Fever	Fever	Communicable
12	Fever with other symptoms not listed above	Fever with other symptoms not specified above	Communicable
13	Fractures	Fractures	Injury
14	High blood pressure (≥140/90)	High blood pressure	Non-communicable
15	Known diabetes	Known diabetes mellitus	Non-communicable
16	Open wounds and bruises/burns	Open wounds and bruises/burns	Injury
17	Skin disease	Skin disease	Communicable
18	Fever with headache, muscle pains and any of the following: eye irritation, jaundice, skin rash, scanty urination	Suspected leptospirosis	Communicable
19	Fever with rash	Suspected measles	Communicable
20	Fever with severe headache and stiff neck in children 12 months and older/Fever and bulging fontanels or refusal to suckle in children <12 months	Suspected meningitis	Communicable
21	Spasms of neck and jaw (lock jaw)	Tetanus	Communicable

From Health Emergency Management Bureau (3).

SPEED uses data gathered by public health facilities including evacuation centers, village health centers, community health centers, and hospitals. Each SPEED report includes the number of cases for each of the 21 syndromes monitored by the system as implemented by HEMB. In 2013, reporting health facilities transmitted data on a daily basis by courier, Short Message System (SMS), or via Internet. Patients’ names, age, and gender were not entered into the database (3).

For the purpose of this study, we were granted access to the database by HEMB. Since this analysis is based on secondary anonymized data sets, further data anonymization and ethical approval were not required. All SPEED database reports were handled with confidentiality by the authors.

Syndrome rates were calculated as the crude rate of the total consultations for a syndrome or syndrome group divided by the total population in the catchment area of the reporting health facility. Population data were derived from the 2010 Census of the Philippine Statistics Office (21). Municipality or city population data were used for community health centers and hospitals, while village populations were used for evacuation centers and village health centers. We assumed the population data used for evacuation centers to be equivalent to the corresponding village, but there might be cases in which the catchment areas were bigger or smaller than the actual village.

The analysis was limited to 150 days post-disaster, since there were only 12 SPEED reports from the flood that exceeded this time period. Syndrome rates and corresponding 95% confidence intervals as well as *t*-tests comparing rates between time periods (within 2 months versus after 2 months post-disaster) and between disaster types were computed using STATA (22).

## Results

**Fig. 1:**
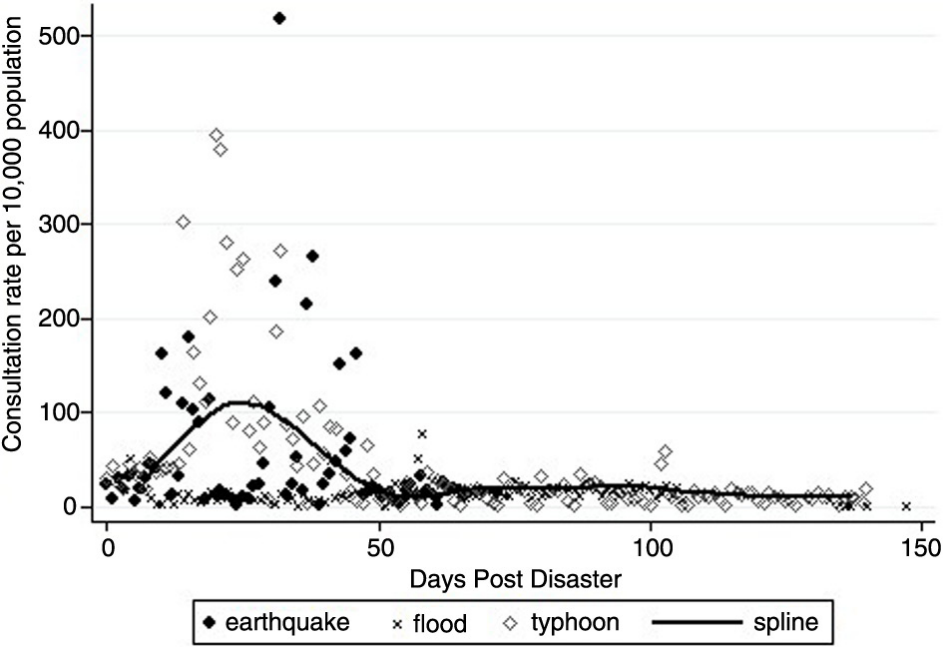
Consultations rates per 10,000 individuals for the earthquake, flood, and typhoon.

For all three types of disasters a total of 4,645 SPEED reports was available; typhoon, earthquake, and flood accounted for 3,425, 609, and 611 reports, respectively. Total syndrome rates were 59.2 (95% CI: 50.6–67.8), 68.2 (95% CI: 46.1–90.2), and 20.7 (95% CI: 15.9–25.4). Around day 50, there was a decrease in the consultations and variability of syndrome rates for the typhoon. After the earthquake, there was a high variability of consultations from day 1 post-disaster to day 46. The scatter plot of the flood showed little variability in consultation rates over time (see Fig. 1).

Compared with the earthquake and the flood, the typhoon had the highest consultation rates in all syndrome groups for the first 2 months post-disaster [communicable diseases, 84.5 (95% CI: 69.5–99.4); injuries, 10.9 (95% CI: 8.3–13.5); and NCDs, 10.3 (95% CI: 8.4–12.2)]. While there were more injuries than NCDs after the flood, it was vice versa after the earthquake. Both syndrome groups showed similar rates after the typhoon. Two months post-disaster, however, all three disasters displayed almost identical consultation rates. Consultation rates for communicable diseases were in the range of 14.2 to 14.7, while consultation rates for injuries and NCDs were below 3 per 10,000 individuals. Only Typhoon Haiyan showed significant differences comparing rates within 2 months and after 2 months post-disaster, including the observation of *p*-values below 0.05 (see Table 2).

**Table 2:** Syndrome rates per 10,000 individuals with 95% confidence intervals disaggregated by syndrome group comparing within 2 months and after 2 months post-disaster

Disaster	Communicable diseases	Injuries	Non-communicable diseases
≤2 months post-disaster			
Flood (*N*=551)	18.8 (14.2–23.5)	1.7 (0.9–2.5)	0.7 (0.5–1.0)
Earthquake (*N*=582)	54.9 (36.5–73.2)	6.5 (4.3–8.6)	9.2 (5.2–13.1)
Typhoon (*N*=1,614)	84.5 (69.5–99.4)	10.9 (8.3–13.5)	10.3 (8.4–12.2)
>2 months post-disaster			
Flood (*N*=60)	14.5 (12.4–16.7)	0.4 (0.1–0.6)	0.2 (0.1–0.2)
Earthquake (*N*=27)	14.7 (11.3–18.2)	1.2 (0.6–1.7)	1.5 (0.7–2.3)
Typhoon (*N*=1,811)	14.2 (12.5–15.8)	1.4 (1.1–1.7)	2.3 (1.9–2.6)
Difference between ≤2 months and >2 months post-disaster			
Flood	4.3 (*p*=0.55)	1.3 (*p*=0.28)	0.5 (*p*=0.15)
Earthquake	40.2 (*p*=0.36)	5.3 (*p*=0.30)	7.7 (*p*=0.41)
Typhoon	70.3 (*p*<0.01)	9.5 (*p*<0.01)	8.0 (*p*<0.01)

The most common syndromes for all three syndrome groups were acute respiratory infections (ARIs), wounds, and high blood pressure. The top six syndromes for all disasters combined were ARIs [32.9 (95% CI: 28.2–37.6)], open wounds, bruises, and burns [5.2 (95% CI: 4.3–6.2)], high blood pressure [4.6 (95% CI: 3.8–5.3)], skin disease [4.1 (95% CI: 3.4–4.8)], fever [3.0 (95% CI: 2.4–3.5)], and acute watery diarrhea [2.2 (95% CI: 1.9–2.5)]. The difference between rates within and after 2 months for the said syndromes are the following: ARIs (37.4, *p*<0.01), open wounds, bruises, and burns (6.5, *p*<0.01), high blood pressure (4.8, *p*<0.01), skin disease (5.1, *p*<0.01), fever (3.9, *p*<0.01), and acute watery diarrhea (2.6, *p*<0.01).

## Discussion

Communicable diseases were by far the most common group of syndromes observed for all three disasters. According to the literature on the health impact of disasters, infectious diseases manifest after natural hazards (regardless of hazard type) due to health system vulnerability and the disruption of basic needs. Basic services, such as adequate water and sanitation, proper shelter, as well as health services, are lacking due to the disaster. Moreover, infectious diseases observed in previous disasters were often endemic in the affected areas (11, 13). Our study results suggest communicable diseases are the most common syndrome group regardless of hazard type.

The top six syndromes, seen for all three disasters combined, reflected the morbidity profile of the country. The top 10 causes for morbidity included respiratory conditions such as ARIs, pneumonia, bronchitis, fever (represented in the form of influenza), tuberculosis, acute watery diarrhea, injuries, and hypertension (23).

Injuries were attributed mostly to earthquakes and typhoons due to situations of falling infrastructure, storm surges, and flying debris (11, 24, 25). In our study, injuries in the form of open wounds, bruises, and burns were the most common among their syndrome group in all three disasters. This pattern of having minor injuries rather than fractures after an earthquake and typhoon may be attributed to the common causes of mortality: drowning in typhoons and major trauma from earthquakes (24, 26). Because most of the local and international responders arrived after 48 h post-disaster from outside the affected region (27, 28), it was to be expected that there would be more mortalities than serious injuries. Thus minor injuries predominated over major injuries such as fractures. This had previously been observed in the Bam Earthquake in Iran (25).

Regardless of the morbidity profile of the regions, a significant amount of patients in all three disasters sought consult for NCDs, hypertension being the most common. This highlights the need for health services addressing NCDs during disaster response and recovery.

So far, there has been no consensus in the literature on when disaster response ends and when recovery begins. According to Runkle et al., the first, the acute response phase, is contained within the first 4 days post-disaster and is followed by the latter, the recovery phase, when the health system has to handle the secondary surge of primary care needs of a population (29). In contrast, we suggest using the disaster risk reduction framework proposed by HEMB to demarcate the two phases according to four thematic areas of disasters: disaster preparedness, disaster response, disaster recovery and reconstruction, and disaster mitigation and prevention.

The periodization of the SPEED reports into within 2 months and after 2 months was reflective of the syndrome patterns in the scatter plots. Our study has shown that after 2 months post-disaster, there was a strong decline in consultations. This decline may allow a rough demarcation between response efforts and recovery. Within 2 months post-disaster, we observed a clear increase of consultation rates for all three disasters (flood<earthquake<typhoon). This observation is further supported by the lack of variability seen in the consultation rates of the flood. We conclude that these differences may be reflective of the severity of the particular disasters and the magnitude of the disruption of the health system. After the 2-month mark, the rates for all three disasters were similar, which may be an indicator for the beginning of the recovery phase. Furthermore, the response syndrome rates also correlate with the estimates of the Sphere Project. The Sphere standards estimate the number of new consultations post-disaster to lie between two and four per person per year (30). If we were to convert this range to a rate per 10,000 individuals per day, it would equal 55 to 110 consultations per day. The rates found in the Sphere standards are applicable only for the response syndrome rates and not beyond that cut-off.

This demarcation of syndrome rates at the 2-month mark has a number of organizational implications such as planning team deployment cycles that are reflective of the expected fatigue of medical staff due to the increasing number of consultations. It also improves the preparation of supplies and medications needed to support the health services during the disaster response within the first 2 months. Because of the evidence from the study, the time of deployment and appropriateness of medical staff could be better planned and more cost-effective interventions could be ensured.

SPEED's role in decision-making as a feedback mechanism during disasters can be broken down using the health system building blocks of WHO. SPEED is a health information system that guides the governance of disasters with regards to human resources, service delivery, and logistics. The health systems approach and all-hazards approach have been suggested as means of achieving resilience in communities in the face of natural hazards and disasters (31).

As an early warning and alert system, SPEED provides real-time information that enables early intervention in outbreaks of infectious disease (3, 6). SPEED has been used in major disasters in the Philippines prior to 2013 with the purpose of detecting outbreaks during disaster response and recovery. However, analyzing the SPEED database post-disaster benefits future disaster responses and planning. Planning logistics and health facility needs based on the collected data will improve disaster preparedness. One of the aims of this study is to guide the logistics management of HEMB and other actors in the humanitarian and disaster management fields. To conform with the Interagency Emergency Health Kits, we present directly applicable syndrome rates per 10,000 individuals (32). The HEMB health kits may also be adapted to the observed rates. Furthermore, SPEED compensates for the weakness of existing surveillance mechanisms that cannot provide the immediate information required for health response needs in disasters and emergencies.

Using the health systems approach in the analysis of SPEED corresponds with previous studies on disasters. Phalkey et al. analyzed a flood from 2008 in India using the health systems approach and suggested improvements in health facilities using the six building blocks (33). WHO has been continually promoting health systems resilience in the Western Pacific region by issuing technical guides that focus on health facility preparedness, assessments on the impact of disasters to health systems, and essential service packages for disasters (34).

The results of this study underline, that the focus in disaster response has to be on the immediate implementation of basic health interventions. For hazards that can be monitored prior to the impact, such as typhoons and floods, HEMB recommends to be prepared for the prepositioning of logistics during the pre-impact phase (2). The syndrome rates of the three disasters showed that the most common syndromes involved in a post-disaster scenario can be met by measures such as providing for shelter, water, sanitation, hygiene, nutrition, and primary health services addressing common communicable diseases, injuries, and NCDs (35).

Since SPEED is a secondary data source, the quality of the data is difficult to assess, even with the initial validation done by HEMB (3). The data is also limited by the fact that the severity of the different conditions has not been entered. This means, for instance, that all wounds, bruises, and burns were recorded without mentioning the necessity for surgery or, in minor cases, wound cleaning. Furthermore, the applied population denominators may only roughly estimate the actual number of individuals within the respective catchment areas, since out-migration of affected people was not taken into account. It cannot be ruled out that there were other outbreaks or spikes in disease prevalence due to the migration of displaced people to another municipality, city, province, or region. However, we expect the true populations, within the affected areas, to be less than the reported statistics from the Philippine Statistics Office in 2010. This may have led to an underestimation of consultation rates.

## Conclusions

Overall, the common disaster syndromes correlate with the country's morbidity profile. Communicable diseases, specifically ARIs, were the most common. Prevention, early diagnosis, and early treatment may reduce these common syndromes. Furthermore, syndrome rates during disaster response depend on the scale of disruption of health systems and hence allow a clear distinction between response and recovery phases. These trends help HEMB and other decision-makers to plan logistics and human resources for future disasters.

## Paper context

Guidelines on priority health conditions in disasters and emergency health kits have been previously established. However, these need further validation using real-world experiences from low- and middle-income countries that are disaster vulnerable. Aside from its early outbreak detection capacity, the real-time surveillance of health effects used in the Philippines, in 2013, has provided a description of the health needs of health facilities and health teams in different hazards affecting the country.
